# Spectral CTA-based quantitative differentiation of infarcted and non-infarcted ASPECTS regions in acute stroke

**DOI:** 10.1186/s41747-026-00733-y

**Published:** 2026-05-13

**Authors:** Schekeb Aludin, Agreen Horr, Patrick Langguth, Tristan Klintz, Svea Seehafer, Alexander Gless, Karim Mostafa, Olav Jansen, Naomi Larsen, Lars-Patrick Schmill

**Affiliations:** Clinic for Radiology and Neuroradiology, University Hospital Schleswig-Holstein Campus Kiel, Kiel, Germany

**Keywords:** Acute ischaemic stroke, ASPECTS, Computed tomography angiography, Infarction (middle cerebral artery), Tomography (x-ray computed)

## Abstract

**Objectives:**

Spectral computed tomography (CT) enables material decomposition and monoenergetic imaging, improving infarct-estimation on non-contrast cerebral CT (NCCT). However, beyond NCCT, infarct estimation is also accessible from CT angiography (CTA). We evaluated the potential of spectral CTA to assess quantitative differences of infarcted parenchyma in patients with stroke.

**Materials and methods:**

In 14 patients with acute middle cerebral artery stroke, the ten Alberta stroke program early computed tomography score (ASPECTS) regions of the affected and non-affected sides were segmented on NCCT, CTA, CT perfusion-derived cerebral blood flow (CBF) map, and CTA-based spectral maps: virtual-non-contrast, virtual-monoenergetic 40-keV and 100-keV, iodine-density and electron-density. For each region, a ratio to the corresponding contralateral region was calculated. Classification into infarcted and non-infarcted was based on post-thrombectomy follow-up NCCT. On patient-level the groups were summarised with mean ratio-values for infarcted and non-infarcted parenchyma per patient. Conventional images and spectral maps were compared to differentiate the groups.

**Results:**

Except for NCCT, electron-density and virtual-non-contrast maps, there was a significant difference between the ratio-values of infarcted and non-infarcted regions in each spectral map, as well as in CTA and CBF (*p* < 0.001). Best differentiation was possible with iodine-density (area under the receiving operating characteristic curve [AUROC] = 1.000) and virtual-monoenergetic 40-keV (AUROC = 0.985) maps compared to NCCT, CTA, CBF, electron-density, and VNC maps (AUROC = 0.765‒0.938).

**Conclusion:**

Spectral CTA maps, like virtual-monoenergetic 40-keV and iodine-density, enabled valid quantitative ASPECTS-based infarct-estimation in stroke compared to conventional NCCT, CTA, and CBF. They harbour potential for improving stroke imaging diagnostics.

**Relevance statement:**

Quantitative values derived from ASPECTS regions in spectral CTA maps improve differentiation of infarcted and non-infarcted regions compared to conventional images. This technique, therefore, represents an interesting approach to improve diagnostic accuracy in stroke and could potentially assist in making treatment decisions for patients with acute stroke.

**Key Points:**

- In patients with acute stroke, spectral CT improves infarct estimation by NCCT, while its benefit with CTA remains unclear.
- Quantitative parameters derived from ASPECTS-labelled region of interest were compared between spectral CTA maps and conventional NCCT, CTA, and CBF.
- Quantitative parameters from virtual-monoenergetic 40-keV and iodine-density maps reliably differentiate infarcted *versus* non-infarcted ASPECTS regions compared to conventional images.

**Graphical Abstract:**

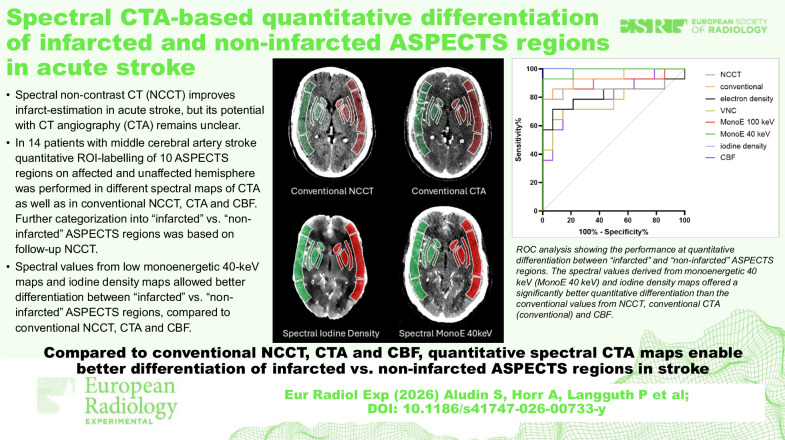

## Background

Acute ischaemic stroke (AIS) is one of the most common neurological emergencies and remains a leading cause of death and long-term disability worldwide [1–3]. Rapid and accurate diagnosis is essential to initiate treatment and thus improve patient outcomes [3, 4]. Although magnetic resonance imaging (MRI) is considered the reference standard for the detection of cerebral ischaemia, it is not widely available in emergency settings and has limitations such as long examination times and exclusion criteria, such as electrical implants [4–7]. Therefore, multimodal computed tomography (CT) imaging consisting of a non-contrast cerebral computed tomography (NCCT), a CT angiography (CTA) of the intracranial arteries, and a CT perfusion (CTP) of the brain parenchyma is the primary acute diagnostic tool for AIS [3, 6]. It is widely available 24/7 and has been evaluated in multiple studies for the valid assessment of the extent and progression of the infarction [3, 6, 7].

In AIS imaging, an important diagnostic criterion is the assessment of infarcted parenchyma. In clinical practice, this is usually derived from NCCT, in which infarcted brain parenchyma shows visually reduced corticomedullary differentiation, oedema and an overall hypodense appearance [5–7]. Scoring systems such as the “Alberta Stroke Program Early CT Score” (ASPECTS) are also used here, in which defined regions of the middle cerebral artery (MCA) territory are visually assessed for the presence of such signs of infarction and the total extent of the infarction is reflected as a score [7–9]. In addition to its application in NCCT, it is also possible to estimate infarct regions in CTA. Although CTA is primarily intended for the assessment of vessels and vascular occlusions, studies have shown that it can also be used to estimate infarcted brain parenchyma, as this shows reduced contrast enhancement and consequently appears relatively hypodense [10, 11]. It even appears that the ASPECTS derived from CTA, referred to as CTA-ASPECTS, is formally more sensitive than NCCT-ASPECTS [10, 12, 13]. Despite this increased sensitivity, however, visual assessment of infarct extent in CTA has not become established in routine clinical practice, most likely due to reasons such as variability in contrast timing or limited image quality for parenchymal evaluation.

However, technical innovations offer new potential in this area. An alternative approach has recently been developed: instead of relying on a purely visual assessment, the estimation of infarcted regions is performed based on HU values measured quantitatively in the brain parenchyma [14–16]. This quantitative approach allows for more objective differentiation of infarct regions, and automated software is increasingly being developed that quantifies brain regions based on region of interest (ROI) and assesses them in regard to infarction [14–18]. Studies on this topic have already been conducted for both NCCT and CTA, and CTA has shown a high diagnostic performance in this regard as well [17–21].

Another interesting development is spectral CT, the clinical application of which has increased rapidly in recent years [22, 23]. Spectral CT enables the detection of multiple x-ray energies and thus applications such as virtual-monoenergetic imaging or material decomposition. These can be used to create special tissue maps, such as iodine density maps, or to improve soft tissue contrast and overall image quality [22, 23]. Studies have shown both visually and quantitatively that spectral maps of NCCT can improve the delineation of infarcts and thus make a positive contribution to the diagnostic workflow in AIS [24–29]. The application of spectral maps in CTA, on the other hand, has so far focused mainly on demonstrating improved vascular imaging, which is also in line with the current clinical application of CTA [25, 30]. Although previous studies have explored CTA-derived spectral parameters for the delineation of ischaemic tissue, these approaches were generally not based on a systematic quantitative evaluation within the ASPECTS topography [31, 32]. Yet, in view of the already improved delineation of infarct regions in conventional CTA and the new possibilities offered by spectral imaging, such as improved image quality or new quantification parameters (*e.g*., iodine concentration), this poses an interesting question that has not yet been systematically investigated.

The present study aims to investigate the extent to which spectral maps of CTA, such as monoenergetic maps or iodine density maps, enable a differentiation between infarcted and non-infarcted brain parenchyma compared to conventional methods such as NCCT, CTA, and CTP. To address this, a quantitative, ROI-based approach will be used, in which the standardised ASPECTS regions in the cerebral parenchyma of the MCA territory are marked as ROIs and quantitative values (*e.g*., HU, iodine concentration, etc.) of the various maps are derived from them. Accordingly, no dedicated ASPECTS scoring will be applied, but instead, the standardised, anatomical ASPECTS system is used as topographical landmarks for standardised ROIs in the MCA territory.

## Methods

### Study design and cohort

We conducted a retrospective, single-centre study with consecutive inclusion of patients who were admitted to our emergency department with AIS in the MCA territory and received multimodal CT imaging with a spectral detector CT equipment, followed by successful interventional thrombectomy from September 2020 to February 2022. This study was conducted in accordance with the tenets of the Declaration of Helsinki and its amendments, with local IRB approval (Approval No. D567/18 from 15.11.2018 and its additional amendment from 29.11.2022) and informed patient consent. Diagnostic and therapeutic procedures reflected the standard of care at our hospital.

We intentionally applied explicit inclusion and exclusion criteria to select a methodologically homogeneous and precise study cohort: Inclusion criteria were: (1) confirmed vascular occlusion involving the MCA territory (internal carotid artery, MCA in vascular segments M1 and dominant M2); (2) available initial NCCT (IN-NCCT), CTA, and CTP; (3) available spectral data sets of CTA; (4) available follow-up NCCT (FU-NCCT) acquired between 6 and 24 h after successful recanalisation; (5) and no high-grade (> 70%) stenosis in the contralateral common or internal carotid artery. Exclusion criteria were: (1) artefacts in the CTP dataset due to motion or poor contrast, as indicated by the semi-automated analysis software IntelliSpace® Portal 11 (Philips Healthcare, Best, the Netherlands); (2) presence of haemorrhage, tumour or postischemic lesions in the MCA territory, or embolism in other cerebrovascular territories; (3) thrombectomy performed with an angiographic result of a “modified treatment in cerebral infarction” (mTICI) score less than 3; and (4) ASPECTS according to IN-NCCT and FU-NCCT of 10 and thus no presence of infarcted regions.

### Image acquisition and reconstruction

All patients were examined according to our institution’s standard stroke imaging protocol using a first-generation spectral detector CT scanner (IQon, Philips Healthcare, Best, the Netherlands) equipped with a 4 cm detector and 128 detector rows. Spectral detector CT is based on a dual-layer technology, which uses two detector layers to detect multiple X-ray energies and generate spectral data from each scan. This data can be used for material decomposition and virtual-monoenergetic imaging. All patients underwent multimodal CT imaging consisting of IN-NCCT, CTA and CTP. They also received FU-NCCT as part of their diagnostic work-up between 6 and 24 h after successful interventional recanalisation of mTICI-3.

#### IN-NCCT and FU-NCCT

IN-NCCT and FU-NCCT covered the whole brain, and conventional images with a slice thickness of 0.8 mm were reconstructed. The technical scan parameters are given in Table 1.

**Table 1:** CT scan technical parameters

Technical parameters	Non-contrast CT	CTA	CT perfusion
Tube voltage	120 kVp	120 kVp	120 kVp
Pitch	0.39	1.046	1.046
Gantry rotation time	0.4 s	0.4 s	0.4 s
Scan time	4.4 s	4.4 s	58 s
Collimation	64 × 0.625	64 × 0.625	64 × 0.625
Scan delay	‒	4 s	5 s
Volume of contrast agent	‒	60 mL	40 mL
Infusion speed	‒	5 mL/s	4 mL/s
Number of cycles	‒	‒	17
CTDI_vol_	38.6 mGy [38.6–38.6]	4.6 mGy [4.18–5.93]	3.6 mGy [3.6–3.6]
DLP	695.6 mGy × cm [662.4–715.7]	183.4 mGy × cm [162.68–241.85]	489.6 mGy × cm [489.6–489.6]

Values for CTDIvol and DLP are reported as median [interquartile range]. For non-contrast CT and CT perfusion, CTDI/Vol values were constant across all examinations due to fixed acquisition settings (for CT perfusion CTDI/Vol value per cycle is given). For CT perfusion, DLP values were constant across all examinations due to fixed acquisition settings. Interindividual variability of CTDIvol was observed for CT angiography due to automated tube current modulation

*CT* Computed tomography, *CTDIvol* CT dose index volume, *DLP* Dose-length product

#### CTA of the intracranial arteries

Iodinated contrast agent (Iomeron® 350 (active molecule: Iomeprol), Bracco, Milan, Italy) was used for monophasic, arterial phase CTA of the intracranial arteries. Bolus-tracking was performed with a threshold of 150 HU in the ascending aorta and with a fixed scan delay after reaching the threshold. The CT scan started at the upper end of the aortic arch and proceeded caudocranially to the vertex. The detailed technical scan parameters are given in Table 1. Conventional images were reconstructed with a slice thickness of 1 mm. Spectral base image datasets were also generated and used to derive the following spectral maps, each with a slice thickness of 1 mm: virtual-non-contrast (VNC), virtual-monoenergetic images at 40 keV (MonoE 40 keV) and 100 keV (MonoE 100 keV), iodine density and electron density.

#### CTP

CTP was assessed using the “toggling-table-technique” with a second intravenous infusion of iodinated contrast agent (Iomeron® 350 (active molecule: Iomeprol), Bracco, Milan, Italy). The technical scan parameters are given in Table 1. The total *z*-coverage of the scan was 8 cm to cover the supratentorial brain parenchyma, and eight slices of 10-mm thickness were reconstructed, which reflects routine clinical workflow. The cerebral blood flow (CBF) map was used for image analysis, as it is considered a suitable parameter for assessing irreversibly infarcted parenchyma, and is frequently used in clinically applied automated perfusion analysis software [33, 34]. The CBF maps were calculated using the deconvolution-based perfusion analysis application implemented in IntelliSpace® Portal 11 (Philips Healthcare, Best, the Netherlands). Semi-automated analysis was performed with visual control of the correct automated placement of the ROI for the arterial input function (preferably in the contralateral M1-segment; if artefacts or anatomical variants were present, the terminal segment of the internal carotid artery was used as an alternative) and the venous output function (preferably in the superior sagittal sinus; if artefacts or anatomical variants were present, the contralateral transverse sinus was used as an alternative), as well as visual quality control of the derived CBF maps.

### Image reading

#### ASPECTS in FU-NCCT as reference standard

The ASPECTS in FU-NCCT was used as the reference standard for final infarction, in line with standard clinical routine, since MRI scans are not routinely performed and were unavailable in this retrospective study-design. The ASPECTS regions on IN-NCCT, CTA and CBF were categorised accordingly as either infarcted or non-infarcted. ASPECTS scoring in the FU-NCCT was performed by three radiologists (T.K., radiologist with 7 years of experience; S.A., radiologist with 5 years of experience; L.-P.S., radiologist with 4 years of experience in acute stroke imaging) according to the established ASPECTS methodology [8, 9]. The raters were blinded to clinical data other than the infarct side. After individual rating, deviating results were adjusted in a consensus reading session.

#### Quantitative measurements of the ASPECTS regions in spectral maps and conventional images

**Fig. 1:**
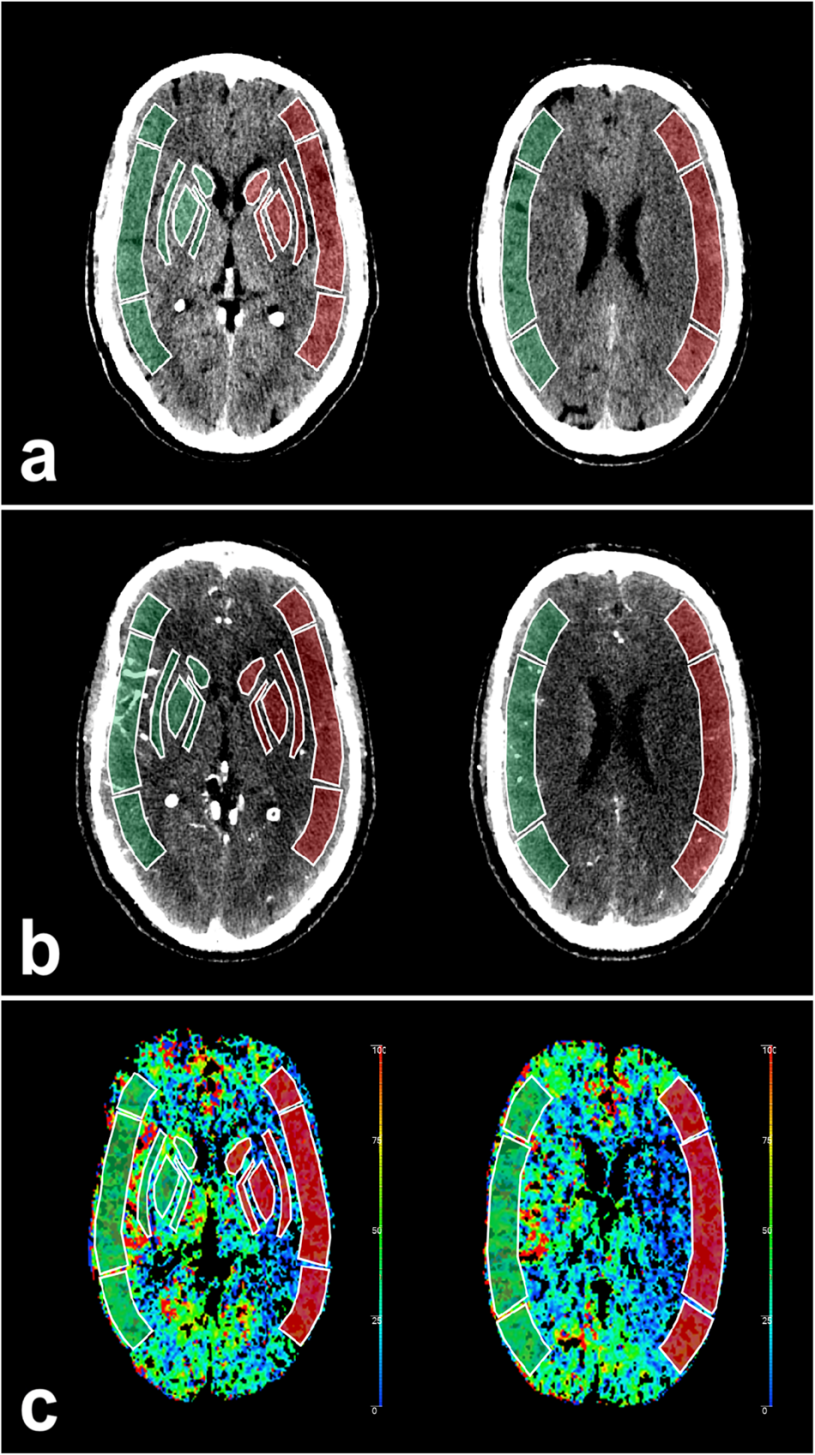
**a** Initial non-contrast CT (window level/width in HU = 30/40). **b** CTA (window level /width in HU = 60/90). **c** CT perfusion-derived CBF map (scale bar added with mL/100 g/min CBF) of an individual with acute infarction in the cerebral territory of the left MCA. The ASPECTS regions on both the affected side (red) and the non-affected side (green) are marked in the different imaging modalities. ASPECTS, Alberta stroke program early computed tomography score; CT, Computed tomography

**Fig. 2:**
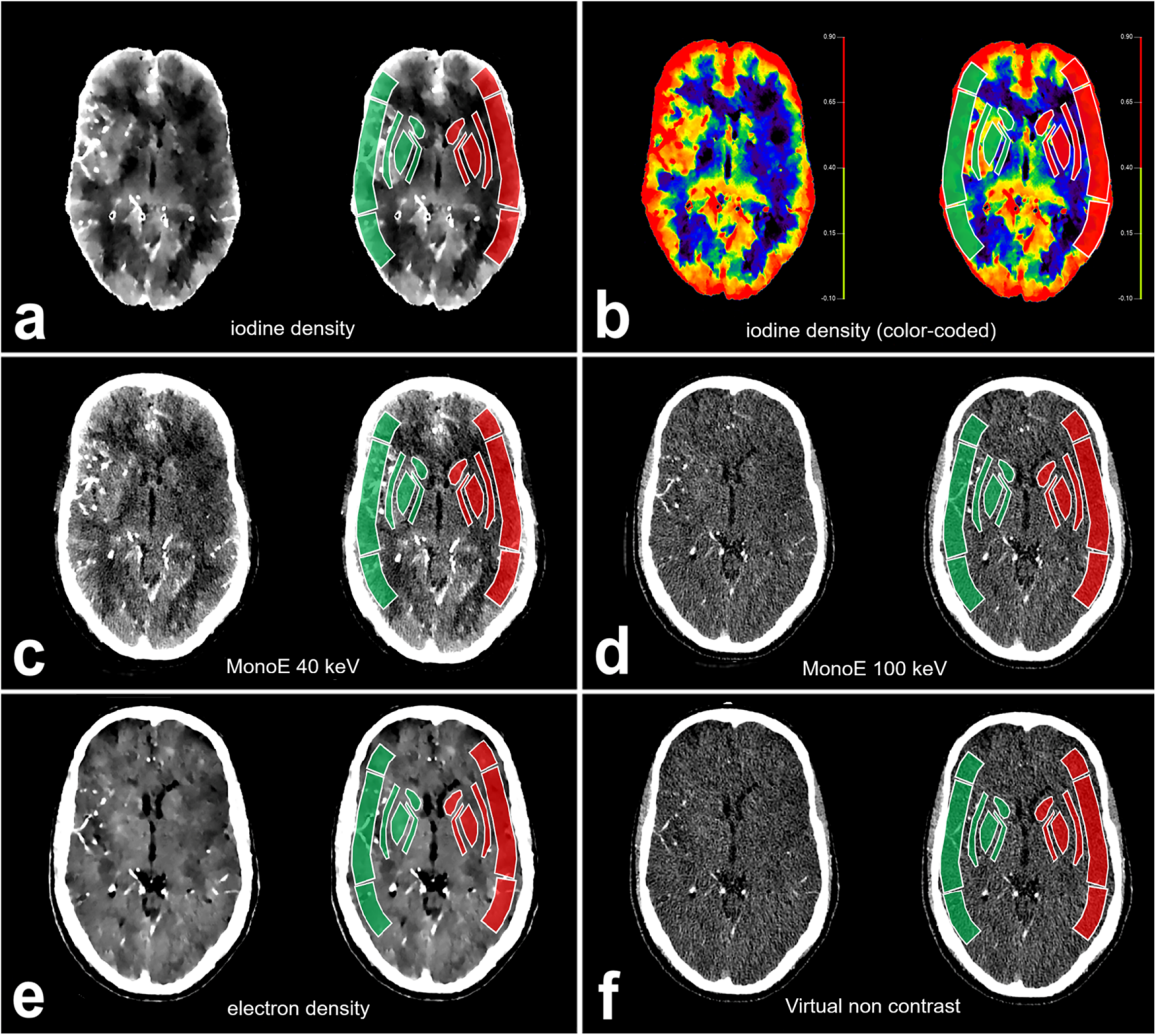
Different spectral maps derived from CTA at the level of the basal ganglia in a patient with an infarct-affected region in the left MCA territory. **a** Iodine density (window level/width in iodine concentration in mg/mL = 0.4/0.8). **b** Iodine density (colour-coded scale in mg/mL iodine concentration). **c** Virtual-monoenergetic images at 40 keV (MonoE 40 keV) (window level/width in HU = 75/120). **d** virtual-monoenergetic images at 100 keV (MonoE 100 keV) (window level/width in HU = 50/90). **e** Electron density (window level/width in relative % electron density = 103/3). **f** Virtual non contrast (window level/width in HU = 50/90). Each panel shows the unlabelled image on the left, while on the right, there is an image with labelled regions of the ASPECTS on both hemispheres (green = non-affected hemisphere; red = affected hemisphere). ASPECTS, Alberta stroke program early computed tomography score; CT, Computed tomography

Quantitative measurements of the ASPECTS regions were performed by two radiologists (S.A. and A.H., radiologist with 4 years of experience in acute stroke imaging) at least four weeks after the ASPECTS scoring was performed. The raters were blinded to clinical data other than the infarct side. The analysis was performed using the manufacturer’s proprietary software, IntelliSpace® Portal 11 (Philips Healthcare, Best, the Netherlands). The ten ASPECTS regions on the affected side and their non-affected counterparts were marked as ROIs on the different imaging maps. In doing so, the raters strictly followed the rules for anatomical landmarks to mark the regions in accordance with the standard ASPECTS methodology (caudate, internal capsule, lentiform nucleus, insular cortex, M1–M6) [8, 9]. In the conventional IN-NCCT and CTA, the Hounsfield unit (HU) values were determined for each ROI (Fig. 1). Accordingly, the regional CBF (in mL/100 g/min) was measured in the CBF map (Fig. 1). In the spectral VNC, the MonoE 40 keV and the MonoE 100 keV maps, the regional HU-values were measured. In the iodine density and the electron density, the regional concentration of iodine (in mg/mL) and the relative electron density (in %) were measured, respectively (Fig. 2).

### Quantitative assessment and statistical analysis

Statistical analysis was performed using GraphPad Prism 10 (GraphPad Software, Boston, USA). Normal distribution was tested with the D’Agostino & Pearson test for all values. The intraclass correlation coefficient (two-way random effects model, absolute agreement) was used for the inter-rater comparison and calculated using Jamovi version 2.3.28 (The Jamovi project, 2023 [www.jamovi.org]). The level of significance was set at 0.05 for all tests.

#### Overall assessment of infarcted and non-infarcted ASPECTS regions

The absolute quantitative values of the ASPECTS regions on the affected side were compared to the corresponding ASPECTS regions on the opposite, non-affected hemisphere. For each region on the affected side, a ratio of the quantitative values was calculated to its contralateral counterpart. Based on the assessment of the ASPECTS regions in the FU-NCCT as either infarcted or non-infarcted, the ASPECTS regions could then be categorised as “infarcted” or “non-infarcted” across all patients. Thus, ratio values for each infarcted and non-infarcted ASPECTS region were derived.

#### Patient-level comparison between infarcted and non-infarcted ASPECTS regions

To achieve standardisation and circumvent intra-patient factors for statistical testing, we performed a patient-level calculation of the ratio of infarcted and non-infarcted regions. ASPECTS-scoring in FU-NCCT provided a group of infarcted and non-infarcted regions on the affected hemisphere in each patient. For each group, the mean values of the quantitative measurements in the respective regions were summarised and a ratio was calculated to the contralateral, corresponding regions on the non-affected hemisphere. Consequently, each patient provided one mean ratio value for the infarcted and one for the non-infarcted regions. These mean ratio values were then compared between NCCT, CTA, CBF and the different spectral maps of the CTA, as well as with the corresponding ratios of the non-infarcted regions, using a two-way repeated measures ANOVA with Šídák’s multiple comparisons test. Furthermore, it was evaluated, which imaging map provided good differentiation between infarcted and non-infarcted regions by using receiver operating characteristic (ROC) analysis and area under the receiving operating characteristic curve (AUROC) calculation.

#### Comparison of specific ASPECTS regions

Besides this, an additional analysis was performed to assess whether the performance of the maps varied depending on the different ASPECTS regions within the MCA territory. However, to account for the possibility that some regions might have too few patients with corresponding infarcts, the regions were combined in groups according to their anatomical region and proximity. The mean ratio values were calculated for combined ASPECTS regions 1–3 (basal regions: caudate, internal capsule and lentiform nucleus); regions 5–7 (inferior cortical regions M1–M3); and regions 8–10 (superior cortical regions M4–M6). ASPECTS region 4 (insular cortex) was considered separately and not combined with other regions. A two-way repeated measures ANOVA with Šídák’s multiple comparisons test was performed on the mean values of the infarcted and non-infarcted groups of the ASPECTS regions, calculated using the above-mentioned ratios.

## Results

### Study cohort

**Fig. 3:**
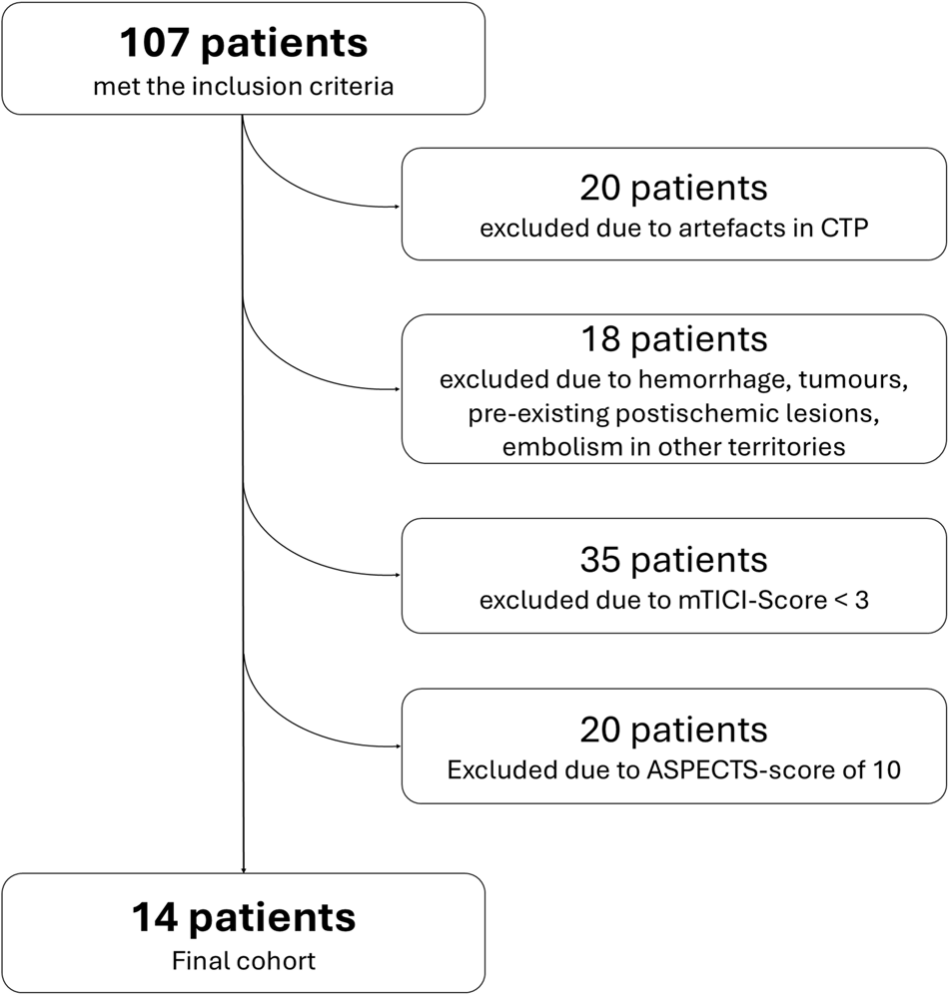
Flow chart of the study cohort, indicating the exclusion criteria and the patients excluded on that basis. ASPECTS, Alberta stroke program early computed tomography score; CT, Computed tomography; CTP, CT perfusion; mTICI, Modified tissue in cerebral infarction (score)

We identified 107 patients who met the inclusion criteria. 20 were excluded due to artefacts in the CTP dataset; 18 were excluded due to the presence of extensive haemorrhage, tumours, pre-existing postischemic lesions in the MCA territory or embolism in other cerebrovascular territories in the IN-NCCT or the FU-NCCT; 35 were excluded due to an angiographic result of an mTICI-Score less than 3; 20 were excluded due to an ASPECTS in IN-NCCT and FU-NCCT of 10. Accordingly, 14 patients met the final inclusion/exclusion criteria (Fig. 3). Basic demographic and clinical data of these patients are given in Table 2.

**Table 2:** Study cohort characteristics

Parameters	Values
Age	75.5 years [57.5–76.25]
Sex	
Men	3
Women	11
Occluded vessel-segment	
Internal carotid artery	3
MCA M1-segment (number of patients)	6
MCA M2-segment (number of patients)	5
ASPECTS score in initial non-contrast CT (NCCT)	7.5 [5.75–8.25]
ASPECTS score in follow-up NCCT	7.5 [5.75–9.0]
Time from symptom onset to initial NCCT^*^	92 min [66–135]
Time from symptom onset to recanalisation^*^	199 min [181–229]
Time from NCCT to CTA	183 s [134.75–245]
Time from CTA to CTP	57.5 s [48.75–72.25]
Time from initial NCCT to recanalisation	87.5 min [77–103.25]
Time from initial NCCT to follow-up CT	464.5 min [347.75–510.25]

Except for the parameters “sex” and “occluded vessel-segment”, data are given as median [interquartile range]

*ASPECTS* Alberta stroke program early computed tomography score, *CT* Computed tomography, *CTA* CT angiography, *CTP* CT perfusion, *MCA* Middle cerebral artery, *NCCT* Non-contrast cerebral CT

^*^ Regarding the data on time from symptom onset to initial NCCT, the specific time of symptom onset was available for seven patients, and the calculated value is from these patients; the rest of the cohort experienced “wake-up-symptoms” without a specific time of symptom onset and therefore could not be included in the calculation

### Quantitative differentiation of infarcted and non-infarcted ASPECTS regions

#### Overall assessment of infarcted and non-infarcted ASPECTS regions

**Fig. 4:**
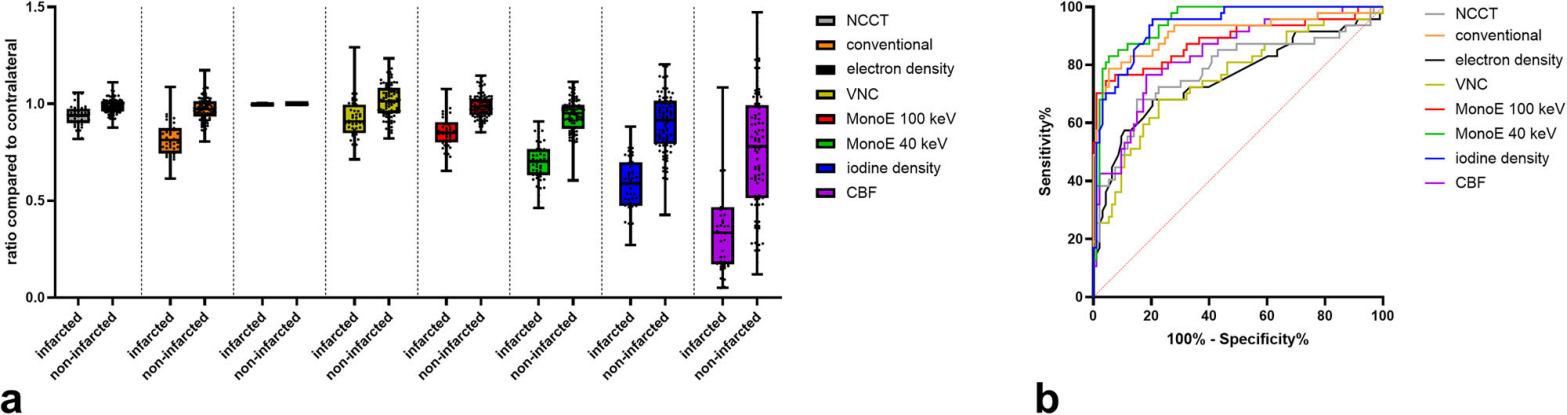
**a** The values of the 140 ASPECTS regions measured in the different spectral maps and modalities were related to the contralateral side to calculate ratios. These ratio values of the groups of final infarcted and non-infarcted ASPECTS regions are given here in a descriptive manner for NCCT, CTA (conventional), electron density, virtual non-contrast (VNC), virtual-monoenergetic images at 100 keV (MonoE 100 keV), virtual-monoenergetic images at 40 keV (MonoE 40 keV), iodine density, and CBF. Boxes show the median as a horizontal line with the range from the 25th to the 75th percentile. Whiskers show the range from minimum to maximum values. Individual values are depicted as dots. **b** ROC-diagram showing the differentiability between the respective groups of finally infarcted and non-infarcted ASPECTS regions based on the calculated ratio values (*n* = 140) for NCCT, conventional, electron density, VNC, MonoE 100 keV, MonoE 40 keV, iodine density, and CBF. ASPECTS, Alberta stroke program early computed tomography score; CT, Computed tomography

In all patients, a total of 140 ASPECTS regions (10 regions per patient in 14 patients) were evaluated on the infarct side. Of these, 47 were classified as infarcted and 93 as non-infarcted, based on the final infarct expansion in FU-NCCT. The inter-rater reliability of the measurements of quantitative values was very good (intraclass correlation coefficient = 0.811 (95% confidence interval [CI] 0.71–0.881). The calculated ratio values for all 140 ASPECTS regions are shown in Fig. 4a for the descriptive comparison of the different spectral maps and the conventional images. Here, one can see that there is virtually no difference between the ratios of the infarcted and the ratios of the non-infarcted regions for electron density, and only a slight difference for NCCT. At lower keV levels, the difference in the ratios increases, as indicated by MonoE 100 keV and MonoE 40 keV. The ratios appear to reach their maximum difference at MonoE 40 keV, iodine density and CBF. The corresponding ROC curves are shown in Fig. 4b.

#### Patient-level comparison between infarcted and non-infarcted ASPECTS regions

**Fig. 5:**
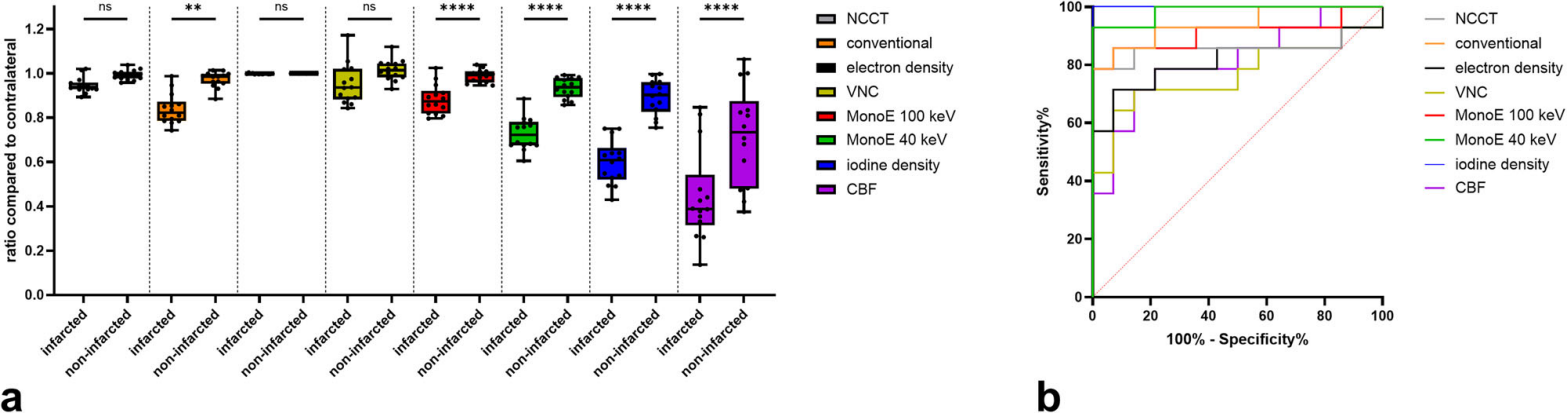
**a** The values of the ASPECTS regions measured in the different spectral maps and modalities on patient-level (*n* = 14) were related to the contralateral side to calculate ratios. These ratio values were compared between the groups of final infarcted and non-infarcted ASPECTS regions in non-contrast-CT (NCCT), CTA (conventional), electron density, virtual non contrast (VNC), virtual-monoenergetic images at 100 keV (MonoE 100 keV), virtual-monoenergetic images at 40 keV (MonoE 40 keV), iodine density, and CBF. The boxes show the median as a horizontal line with the range from the 25th to the 75th percentile. Whiskers show the range from minimum to maximum values. Individual values are depicted as dots. ns, Not significant; ^*^p < 0.05; ^**^p < 0.005; ^***^p < 0.0005; ^****^p < 0.0001. **b** Receiver operating characteristic diagram showing the differentiability between the respective groups of finally infarcted and non-infarcted ASPECTS regions based on the calculated ratio values (*n* = 14) for NCCT, conventional, electron density, VNC, MonoE 100 keV, MonoE 40 keV and iodine density, and CBF. ASPECTS, Alberta stroke program early computed tomography score; CT, Computed tomography

For statistical testing and normalisation, the mean ratios of the infarcted and non-infarcted regions on patient-level in each spectral map and the conventional images were compared using a two-way repeated measures ANOVA, as shown in Fig. 5a. Except for NCCT, ED and VNC, there was a significant difference between these ratio values of infarcted and non-infarcted regions in each spectral map, as well as in CTA and CBF [*F* (1, 208) = 123.9; *p* < 0.001; *p* < 0.001 for each post-hoc comparison except for NCCT, CTA, ED and VNC; NCCT: *p* = 0.821; CTA: *p* = 0.002; ED: *p* > 0.999; VNC: *p* = 0.478]. Focusing on the spectral maps, the lowest ratio values (mean ± standard deviation) in the infarcted regions were found in iodine density (0.599 ± 0.101), followed by MonoE 40 keV (0.728 ± 0.074), MonoE 100 keV (0.882 ± 0.067), VNC (0.952 ± 0.090) and ED (0.998 ± 0.002). In the overall comparison, the mean ratio values in the infarcted regions were lowest for CBF (0.446 ± 0.211), followed by iodine density, MonoE 40 keV, conventional CTA (0.839 ± 0.069) and NCCT (0.946 ± 0.036). To compare the differentiability between infarcted and non-infarcted regions based on these ratio values, a ROC-diagram is given in Fig. 5b. AUROC values were calculated and were higher in iodine density (AUROC 1.0; 95% CI: 1.000‒1.000) and MonoE 40 keV (AUROC 0.985; 95% CI: 0.949‒1.000), than CTA (AUROC 0.938; 95% CI: 0.849‒1.000), followed by MonoE 100 keV (AUROC 0.908; 95% CI: 0.781‒1.000), NCCT (AUROC 0.867; 95% CI: 0.707‒1.000), CBF (AUROC 0.811; 95% CI: 0.648‒0.975), electron density (AUROC 0.811; 95% CI: 0.632‒0.990) and VNC (AUROC 0.765; 95% CI: 0.575‒0.956).

#### Comparison of specific ASPECTS regions

The ten ASPECTS regions were grouped into four groups and the mean ratio values of the conventional images and spectral maps for each individual group were compared, as described above: ASPECTS regions 1‒3 (*F* (1, 40) = 84.43; *p* < 0.001); ASPECTS region 4 (*F* (1, 12) = 38.78; *p* < 0.001); ASPECTS regions 5‒7 (*F* (1, 40) = 33.32; *p* < 0.001); ASPECTS regions 8‒10 (*F* (1, 40) = 47.75; *p* < 0.001). A similar trend to that observed in the overall and patient-level data above can be seen in all groups (Supplementary Material Fig. S1). Hereby, the MonoE 40 keV and iodine density ratios were significantly lower in the infarcted regions than in the non-infarcted regions in all groups, providing good differentiation.

## Discussion

This study demonstrates for the first time that the use of spectral image maps of monophasic CTA, such as the MonoE 40 keV and iodine density maps, can be used to quantitatively differentiate between infarcted and non-infarcted ASPECTS regions in the parenchyma of the MCA territory. Hereby, these maps show very good differentiation compared to conventional images, such as NCCT, CTA and CBF.

Previous studies have shown that spectral CT can aid in infarct assessment in patients with AIS in the context of multimodal CT imaging [24, 26–30, 35–38]. However, these studies have mainly focused on spectral analysis of NCCT. For instance, some studies have created and analysed material decomposition maps from NCCT, which have been demonstrated to more accurately depict parenchymal oedema in infarction [24, 27, 28, 38]. Others have used virtual-monoenergetic maps of NCCT at different energy levels to evaluate which level best delineates oedema and loss of corticomedullary differentiation in infarcted parenchyma [26, 29, 35, 37]. These previous studies suggest that the use of spectral maps from NCCT may improve infarct delineation compared to conventional imaging. However, only a few of them used standardised anatomical regions, such as the ASPECTS regions for assessment, focusing mainly on visual assessment rather than ROI-based quantification [27, 37]. Consequently, quantitative ROI-based approaches remain underrepresented.

Besides NCCT, CTA is also a substantial part of multimodal stroke CT. Although studies on the spectral analysis of CTA data are also available, they mostly concentrate on improving vascular assessment or image-quality [39, 40]. Only a few studies have focused on evaluating the brain parenchyma using spectral CTA. For example, some studies have evaluated a spectrally reconstructed NCCT from the spectral CTA data as a VNC map for assessing brain parenchyma [41–43]. Such VNC maps have been visually compared with NCCT, and studies to date suggest that it is possible to discriminate infarcted tissue, as well as with NCCT [41–43]. In the present study, a VNC map was generated and quantitatively compared with conventional NCCT, using the anatomical framework of the ASPECTS system. However, the quantitative differentiability of infarcted regions in the VNC map was somewhat worse compared to NCCT in our study. One reason for this discrepancy could be that the previous studies used a significantly different analysis method, namely, visual assessment instead of quantitative measurement [41–43]. Visual assessment is more in line with the standard for ischaemia diagnosis, as this is how it is assessed in routine clinical practice. However, it is also significantly more subjective than quantitative measurement and depends on other factors such as image quality. Furthermore, another possible reason for this discrepancy may be due to technical differences during data acquisition. For instance, we analysed data from a spectral detector CT, whereas the previous studies mainly used dual-source scanners from other manufacturers [41–43]. Dual-source scanners use a different spectral technique and algorithm for image acquisition and reconstruction, which could affect the VNC maps derived and thus explain the discrepancy [22, 44].

Direct estimation of infarct size from conventional CTA (and not by conventional NCCT or a spectral VNC map) has been investigated in previous studies as a method known as CTA-ASPECTS [10, 12, 13, 20, 21]. These studies investigated whether density differences in the parenchyma of infarcted and non-infarcted regions in CTA allow for a more accurate assessment than in NCCT. The studies applied the ASPECTS-region system and assessed the regions either visually or in a quantitative ROI-based approach with HU values, suggesting a better performance of CTA-ASPECTS compared to NCCT-ASPECTS [10, 12, 13, 20, 21]. From a quantitative viewpoint, this is consistent with the results of the present study, as shown by the direct comparison between NCCT and conventional CTA regarding the differentiation of infarcted and non-infarcted regions. Accordingly, these prior studies already demonstrated that contrast-enhanced techniques may have enormous potential in differentiating infarcted parenchyma.

Despite the good performance of CTA in estimating infarcted regions, the extent to which spectral CT and analysis may even further improve the differentiation of infarcted regions (aside from using a VNC map) remains insufficiently investigated. Previous studies have demonstrated improved delineation of infarcted areas using spectral CTA. However, these studies did not apply a topographic assessment within the ASPECTS framework and were primarily aimed at deriving perfusion-related parameters, with CTP used as a reference standard [31, 32]. Therefore, the present study addresses an important scientific gap, as spectral CT with monoenergetic imaging and material decomposition shows innovative potential in this area. Analysis of the CTA-derived spectral maps showed good quantitative differentiation between infarcted and non-infarcted ASPECTS regions in MonoE 40 keV and iodine density. These maps either image iodine specifically or attenuate it close to its K-edge, thereby reflecting very well the reduced parenchymal iodine enhancement as an important feature in infarcted brain tissue. In this initial study, these maps were also formally superior to NCCT, conventional CTA and CBF. So, according to our results, it is possible to improve the performance of CTA-based quantitative estimation of infarcted ASPECTS regions by using them.

This study has some limitations. The most significant of these is the relatively small number of patients. This is primarily due to the rather strict inclusion and exclusion criteria applied in this study. However, these criteria were deliberately narrowed to generate a patient cohort that was as precise as possible, tailored to the specific scientific question being investigated. Only patients with an mTICI score of 3 were included in the study to prevent incomplete recanalisation from leading to progressive infarct development, which would ultimately compromise the methodological quality of the FU-NCCT as the reference standard. Furthermore, patients with an ASPECTS of 10 were also excluded, as they had no infarcted regions and therefore no quantitative values for infarcted parenchyma could be derived from them. This would not be in line with the study question. Our strict approach made it possible to generate a highly homogeneous patient cohort, which enabled a precise methodological evaluation of the study question. Nevertheless, it should be noted that this approach limits the generalisability of the results to a broader clinical population to a certain degree at this point. In this context, the present study should be understood as an initial exploratory study of this innovative approach. Further evaluation of the method and its generalisation to a broader patient population should be conducted in larger, primarily prospective studies.

Another limitation is the retrospective nature of the study, meaning it is not possible to control clinically relevant time intervals, such as the time between initial imaging and thrombectomy. As time passes, progressive infarction of the penumbra occurs, which could lead to a discrepancy between the initial multimodal CT assessment and the FU-NCCT selected as a reference. In our study, however, we used an appropriate and realistic time interval between IN-NCCT and recanalisation (Table 2) as part of our clinical standards, so this aspect was considered acceptable as part of routine clinical conditions. Furthermore, by including only mTICI-3 recanalised patients, it was ensured that there would be as little significant progression of the infarction as possible from the time of recanalisation, which could have been the case with only partial recanalisation. Despite the retrospective design, this minimised the potential for discrepancies in the delineated brain parenchyma between the initial imaging and the FU-NCCT.

Furthermore, the categorisation of the ASPECTS regions was based on the demarcated regions in the FU-NCCT, which was used as the reference standard. However, it could be argued that the categorisation should have been based on MRI with diffusion-weighted and T2-weighted sequences instead, as this is considered the most sensitive imaging method for infarcts [3, 5, 7]. Nowadays, however, MRI is not always performed in the acute phase or for follow-up imaging of AIS due to logistical reasons (limited 24/7 availability, longer examinations, contraindications for examinations, like implants), depending on the country and region. In this regard, and given the retrospective nature of the study, MRI was not available as a reference method. Nevertheless, FU-NCCT is an established alternative that is widely used in clinical practice and has been well validated for use in AIS imaging [3, 5, 7, 45]. Accordingly, it represents an adequate alternative that is well-suited for exploratory studies with a retrospective design.

Further limitations arise from the methodology used to acquire the images. We only used one spectral CT technique, namely spectral detector CT. Although this technique provides a very good temporal and spatial resolution of spectral data, it inherently restricts the transferability of the results to systems that use different spectral techniques [22, 44]. However, it should be noted that using only one type of device results in a high degree of technical consistency in the examined data sets regarding acquisition parameters and reconstruction technology. This prevents potential bias due to different acquisition techniques and devices, and therefore also represents a strength of this study.

It should also be noted that we only used monophasic CTA examinations for quantification in this study, as this is the clinical standard for multimodal CT imaging [3, 4, 7]. These examinations are susceptible to haemodynamic variability between different patients, which can limit the validity of the measurements in principle. However, to minimise this influence as much as possible, we used a clinically standardised CTA protocol with bolus tracking and standardised technical parameters for all patients. Additionally, the measured quantitative parameters were compared to the corresponding reference regions on the contralateral hemisphere to compensate for interindividual differences. This approach has also been used in previous studies investigating parenchymal changes in conventional monophasic CTA, as well as general perfusion imaging, and thus represents an established methodology in this field [20, 33, 34]. Still, this approach may also be limited in patients with severe parenchymal defects or pre-existing vascular damage. However, it proved robust in the present study cohort and should be evaluated further in future studies.

Finally, it should be noted that no clinical functional parameters of the patients were recorded or correlated in this study, meaning the scope for assessment in this regard is limited. However, within the framework of the study design, the focus of the study was deliberately placed on the primary methodological evaluation of the spectral differentiability of infarcted brain regions using spectral maps of CTA. Rather than assessing the influence of this method on clinical decision-making processes and patient outcomes, the aim was to evaluate its feasibility as a promising, novel approach. Given the good performance of spectral CTA maps in differentiating infarction demonstrated in this study, future studies with larger patient numbers and a prospective study design could extend the methodology to a broader patient population and incorporate clinical parameters to a greater extent.

The results of our study suggest some interesting potential clinical applications. For example, the number of spectral CT scanners used in clinical practice is increasing, and the automated reconstruction of spectral maps from CTA data simplifies their availability in the acute setting [22, 44]. The formal improvement in the differentiation of infarcted and non-infarcted regions in these maps could be an aid in cases where assessment of infarction in conventional images proves difficult. Furthermore, software for an automated quantitative calculation of infarcted parenchyma or ASPECTS-scoring is being used increasingly in clinical practice. In such cases, ASPECTS regions are scored using a quantitative approach by applying HU-based thresholds for healthy regions on NCCT or CTA, for example [17–19, 46, 47]. As infarcted and non-infarcted ASPECTS regions can be very accurately differentiated in the MonoE 40 keV and iodine density maps of CTA, an automated software quantification based on these maps and parameters could provide a better performance in the assessment and thus in the acute setting. Therefore, spectral CT may be an important tool for further automating the evaluation of patients with AIS and in helping to speed up and improve therapeutic decisions.

In conclusion, our results demonstrate the technical feasibility and quantitative precision of spectral CTA maps, such as MonoE 40 keV and iodine density, in differentiating infarcted from non-infarcted ASPECTS regions, in comparison to conventional imaging techniques like NCCT, CTA and CBF. However, this does not apply to the spectral VNC map derived from CTA, as this map performed worse than conventional imaging techniques in this study. While these results suggest that these maps perform better than the conventional modalities to some extent, it should be noted that this is a first proof-of-concept study of this innovative method. Nevertheless, the results highlight the potential of quantitative spectral CTA analysis as an innovative approach to enhance the existing acute stroke imaging workflow.

## Supplementary information

**Additional File:** **Fig. S1:** The values of the ASPECTS regions measured in the different spectral maps and modalities were related to the contralateral side to calculate ratios. The ratio values were compared between the groups of final infarcted and non-infarcted ASPECTS regions according to the ASPECTS regions groups. Comparison between non-contrast cerebral CT (NCCT), CT angiography (conventional), electron density, virtual non-contrast (VNC), virtual-monoenergetic images at 100 keV (MonoE 100 keV), virtual-monoenergetic images at 40 keV (MonoE 40 keV), iodine density, and cerebral blood flow (CBF) in: (**a**) ASPECTS region 1‒3 (basal regions: caudate, internal capsule and lentiform nucleus); (**b**) ASPECTS region 4 (insular cortex); (**c**) ASPECTS region 5‒7 (inferior cortical regions M1–M3); (**d**) ASPECTS region 8‒10 (superior cortical regions M4–M6). Boxes show the median as horizontal line with the range from the 25th to 75th percentile. Whiskers show the range from minimum to maximum values. Individual values are depicted as dots. ns: not significant; * *p* < 0.05; ** *p* < 0.005; *** *p* < 0.0005; **** *p* < 0.0001.

## Data Availability

The datasets used and/or analysed during the current study are available from the corresponding author on reasonable request.

## Abbreviations

AIS: Acute ischaemic stroke
ASPECTS: Alberta stroke program early computed tomography score
AUROC: Area under the receiving operating characteristic curve
CBF: Cerebral blood flow
CI: Confidence interval
CT: Computed tomography
CTA: CT angiography
CTP: CT perfusion
FU-NCCT: Follow-up non-contrast cerebral computed tomography
IN-NCCT: Initial non-contrast cerebral computed tomography
MCA: Middle cerebral artery
MonoE 100 keV: Virtual-monoenergetic images at 100 keV
MonoE 40 keV: Virtual-monoenergetic images at 40 keV
MRI: Magnetic resonance imaging
mTICI: Modified tissue in cerebral infarction (score)
NCCT: Non-contrast cerebral computed tomography
ROC: Receiver operating characteristics
ROI: Region of interest
VNC: Virtual-non-contrast
