# Supplementary material for: Spectral CTA-based quantitative differentiation of infarcted and non-infarcted ASPECTS regions in acute stroke
Source: Eur Radiol Exp. 2026 May 13;10:64. doi: 10.1186/s41747-026-00733-y (PMC13172188; doi:10.1186/s41747-026-00733-y)
Supplement: Supplementary file 1 — Additional File: Fig. S1: The values of the ASPECTS regions measured in the different spectral maps and modalities were related to the contralateral side to calculate ratios. The ratio values were compared between the groups of final infarcted and non-infarcted ASPECTS regions according to the ASPECTS regions groups. Comparison between non-contrast cerebral CT (NCCT), CT angiography (conventional), electron density, virtual non-contrast (VNC), virtual-monoenergetic images at 100 keV (MonoE 100 keV), virtual-monoenergetic images at 40 keV (MonoE 40 keV), iodine density, and cerebral blood flow (CBF) in: (a) ASPECTS region 1‒3 (basal regions: caudate, internal capsule and lentiform nucleus); (b) ASPECTS region 4 (insular cortex); (c) ASPECTS region 5‒7 (inferior cortical regions M1–M3); (d) ASPECTS region 8‒10 (superior cortical regions M4–M6). Boxes show the median as horizontal line with the range from the 25th to 75th percentile. Whiskers show the range from minimum to maximum values. Individual values are depicted as dots. ns: not significant; * p < 0.05; ** p < 0.005; *** p < 0.0005; **** p < 0.0001. [file 41747_2026_733_MOESM1_ESM.pdf]

Spectral CTA-based quantitative differentiation of infarcted and non-infarcted ASPECTS regions in acute stroke

ELECTRONIC SUPPLEMENTARY MATERIAL

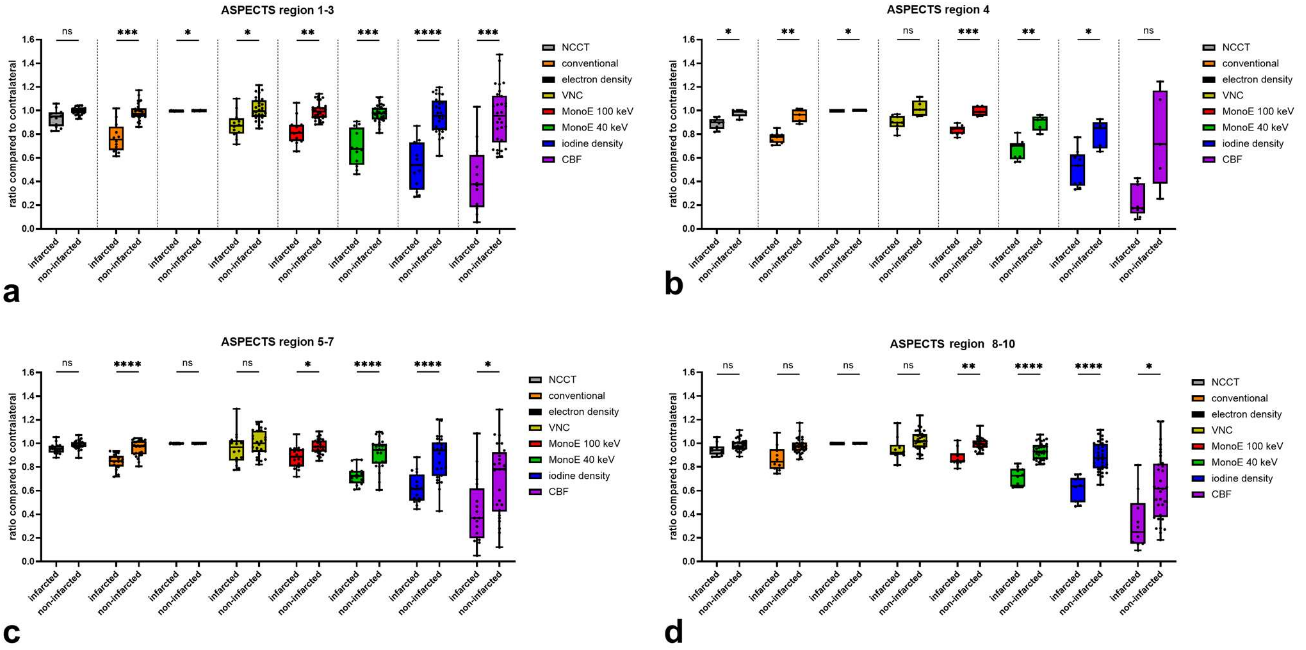

Fig. S1: The values of the ASPECTS regions measured in the different spectral maps and modalities were related to the contralateral side to calculate ratios. The ratio values were compared between the groups of final infarcted and non-infarcted ASPECTS regions according to the ASPECTS regions groups. Comparison between non-contrast cerebral CT (NCCT), CT angiography (conventional), electron density, virtual non-contrast (VNC), virtual-monoenergetic images at 100 keV (MonoE 100 keV), virtual-monoenergetic images at 40 keV (MonoE 40 keV), iodine density, and cerebral blood flow (CBF) in: (**a**) ASPECTS region 1–3 (basal regions: caudate, internal capsule and lentiform nucleus); (**b**) ASPECTS region 4 (insular cortex); (**c**) ASPECTS region 5–7 (inferior cortical regions M1–M3); (**d**) ASPECTS region 8–10 (superior cortical regions M4–M6). Boxes show the median as horizontal line with the range from the 25th to 75th percentile. Whiskers show the range from minimum to maximum values. Individual values are depicted as dots. ns: not significant; * $p < 0.05$; ** $p < 0.005$; *** $p < 0.0005$; **** $p < 0.0001$. *ASPECTS* Alberta Stroke Program Early Computed Tomography Score, *CT* Computed tomography.
